# Feasibility, Efficacy, and Safety of the Mitral Annulo-TRIpsy in eXtreme Risk Patients

**DOI:** 10.1016/j.shj.2025.100683

**Published:** 2025-06-24

**Authors:** Gennaro Giustino, Chantal Y. Asselin, Mostafa Naguib, Ahmad Jabri, Leo Kar Lok Lai, Robert Kipperman, Kostantinos P. Koulogiannis, Leo Marcoff, Amr Abbas, Pedro Villablanca, Philippe Généreux

**Affiliations:** a Gagnon Cardiovascular Institute, Atlantic Health System, Morristown, New Jersey, United States; b Department of Cardiovascular Medicine, Corewell Health William Beaumont University Hospital, Royal Oak, Michigan, United States; c Center for Structural Heart Disease, Henry Ford Hospital, Detroit, Michigan, United States

**Keywords:** Intravascular lithotripsy, Mitral valvuloplasty, Severe mitral annular calcification

## Abstract

**Background:**

Severe calcific mitral stenosis is common and therapeutically challenging. Intravascular lithotripsy (IVL) can facilitate percutaneous balloon mitral valvuloplasty in patients not amenable to conventional therapies. We describe a modified technique using larger IVL balloons to ensure maximal annular contact and delivery of ultrasonic shockwaves to restore mitral leaflet pliability and reduce transvalvular gradients without the need for noncompliant valvuloplasty balloons.

**Methods:**

Seven patients underwent the Mitral Annulo-TRIpsy in eXtreme risk patients (MATRIX) procedure at 3 tertiary structural heart disease centers in the United States. Transcatheter mitral valve replacement was contraindicated due to prohibitive risk of left ventricular outflow tract obstruction or insufficient annular calcification for anchoring of a balloon-expandable valve. IVL balloons were delivered using a large-bore transseptal sheath over three 0.014 wires. Runs of delivery of IVL therapy were repeated until satisfactory results in terms of mean mitral gradient (mMG) reduction were achieved.

**Results:**

Median age was 78 years, and 14.3% were female. All patients presented with progressive New York Heart Association class III-IV symptoms and functional limitations. Pre-MATRIX mMG was 9.0 mmHg. The final mMG was 3.0 mmHg (absolute difference 6.3 mmHg; 95% CI 2.6-10.1 mmHg; *p* <0.01). No conventional valvuloplasty balloons were used after IVL. All patients successfully underwent MATRIX. No major periprocedural complications were observed including death, stroke, major bleeding, or reintervention. No patients experienced worsening mitral regurgitation. All patients were discharged alive.

**Conclusions:**

This small multicenter series demonstrates that IVL of calcified mitral stenosis using the MATRIX technique is feasible and safe and associated with effective reductions in mMG.

## Highlights

• The Mitral Annulo-TRIpsy in eXtreme risk patients (MATRIX) procedure is a viable alternative strategy for intravascular lithotripsy-percutaneous balloon mitral valvuloplasty.
• The MATRIX procedure appears to be effective and safe for severe mitral stenosis due to mitral annular calcification.
• MATRIX reduces transvalvular gradients without increasing mitral regurgitation.
• MATRIX is suitable for high-risk surgical candidates where transcatheter mitral valve replacement is contraindicated.

## Introduction

Severe mitral stenosis (MS) due to mitral annular calcification (MAC) is common and poses a significant challenge to both surgical and percutaneous treatments. MAC is a degenerative process of the fibrous annulus of the mitral valve (MV) that is often asymptomatic and underreported.^1^ The prevalence of MAC is as high as 40% in adults >65 years old.^2^ Most patients with significant MAC are classified as high-surgical-risk candidates due to the presence of multiple comorbidities including advanced age, atherosclerosis, chronic kidney disease, hypertension, and valvular heart disease.^3^, ^4^, ^5^ Furthermore, the presence of severe MAC makes MV surgery technically challenging with an associated 6-fold increase in operative mortality.^6^ Additional studies indicate early mortality rates to be as high as 28% following surgical mitral valve replacement (SMVR) in patients with MAC.^7^^,^^8^ The anatomy of patients with severe MAC is unfavorable for surgery, as the heavily calcified mitral annulus interferes with suture anchoring of the prosthetic valve during SMVR, ultimately increasing the risk of paravalvular leak, atrioventricular groove dissociation, and injury to the left circumflex artery.^9^ Surgeons have adopted modified techniques to facilitate SMVR in patients with MAC to minimize the risk of these complications; however, this requires advanced technical expertise and results in longer cross-clamp and cardiopulmonary bypass times.^6^^,^^10^

Transcatheter mitral valve replacement (TMVR) offers an alternative approach to management for patients at high or prohibitive surgical risk. The majority of TMVR performed in patients with MAC utilizes the SAPIEN 3 valve, which was originally intended for transcatheter aortic valve replacement, as it requires sufficient annular calcification to securely anchor the prosthetic valve. Cardiac computed tomography angiography (CTA) may be utilized to grade MAC severity, predict risk of valve embolization or migration, and determine if TMVR is feasible.^11^ If appropriate, TMVR in MAC remains associated with higher risk for left ventricular outflow tract (LVOT) obstruction, embolic stroke, and perforation.^9^ Unfortunately, patients with MAC are often excluded from TMVR trials. Additionally, patients may not be suitable for TMVR if the MV dimensions are too large for the implantable transcatheter heart valve or if there is too large or insufficient MAC to anchor the valve. Conventional percutaneous balloon mitral valvuloplasty (PBMV) is considered relatively contraindicated in patients with severe MAC or unfavorable Wilkins scores, as it may result in worsening of mitral regurgitation (MR) due to damage to the valvular apparatus.

Intravascular lithotripsy (IVL) is a novel technology for the treatment of calcific atherosclerotic lesions that utilizes acoustic shockwaves via a balloon-delivery system to induce calcium fractures.^12^ Preliminary evidence suggests that IVL can be used to facilitate PBMV with conventional valvuloplasty balloons in patients with severe calcific MS.^13^ The original description of this technique contemplated the use of small shockwave balloons that do not fill the entire MV annulus and may not result in appropriate annular contact to effectively deliver ultrasonic shockwaves, therefore necessitating the use of a standard-sized valvuloplasty balloon. We have previously reported this technique to be feasible in the largest case series of IVL-PBMV for severe MAC in 24 high-risk patients^12^ using combinations of smaller-size IVL balloons followed by final dilatation with a valvuloplasty balloon. Since then, there now exist larger-sized balloons for IVL, which, when used in combination, may achieve greater annular contact and more effective delivery of ultrasonic shockwaves, obviating the use of valvuloplasty balloons.

**Figure 1:**
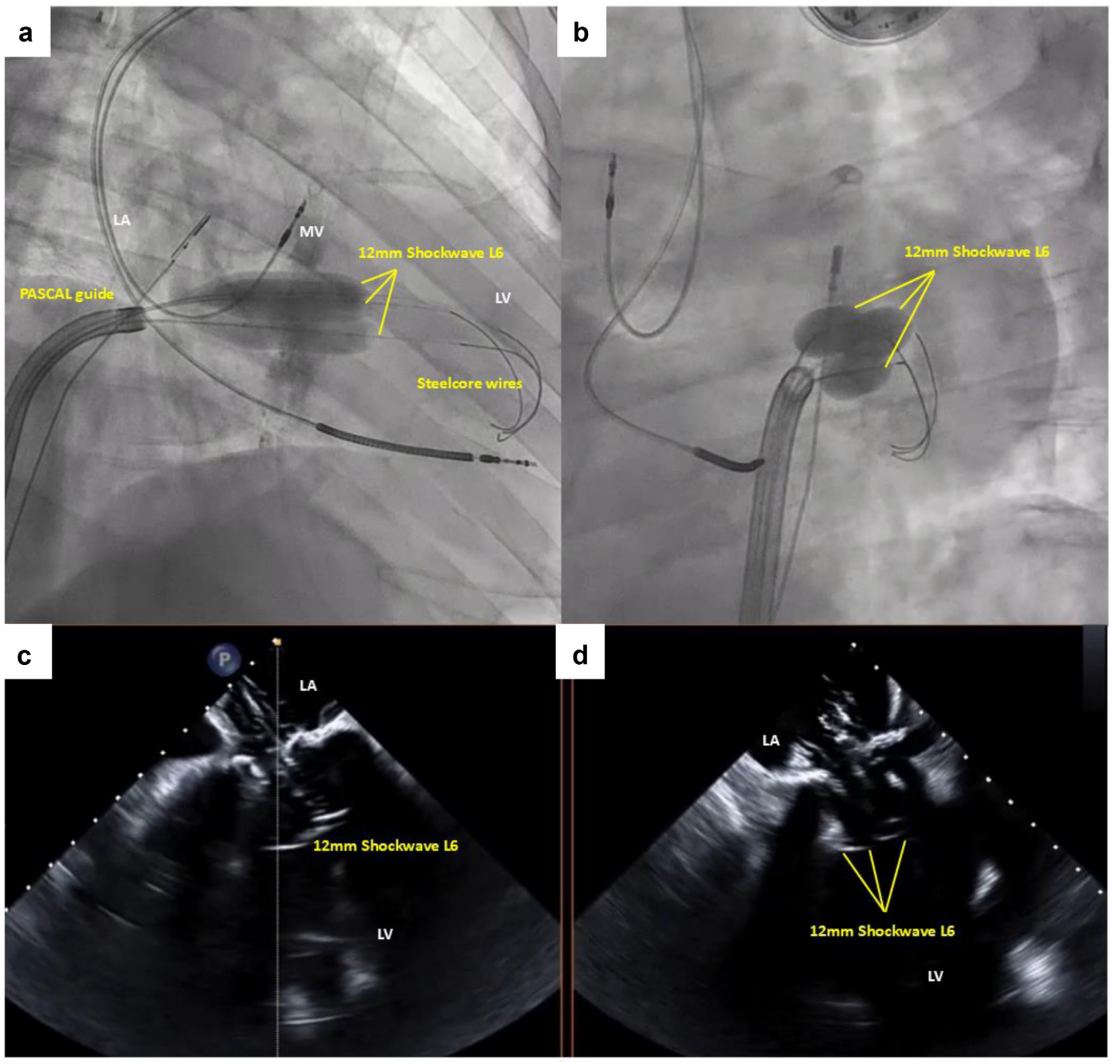
**The MATRIX procedure. (a, b)** Fluoroscopic images demonstrating full annular apposition with 3 × 12 mm shockwave balloons inflated over 3 guidewires. **(c, d)** Transesophageal echocardiographic images demonstrating full annular apposition with 3 × 12 mm shockwave balloons. Abbreviations: LA, left atrium; LV, left ventricle; MATRIX, Mitral Annulo-TRIpsy in eXtreme risk patients; MV, mitral valve.

In this manuscript, we describe the **M**itral **A**nnulo-**TRI**psy in e**X**treme risk patients (MATRIX), which is a modified IVL-PBMV technique to produce lithotripsy of the MV apparatus by oversizing the IVL balloons by 20% in relation to the estimated MV annular size in order to achieve better annular contact and delivery of the ultrasonic shockwaves (Figure 1; Videos 1 and 2). This eliminates the need for standard-sized valvuloplasty balloons, which are noncompliant and may result in worsening of MR or annular rupture.

## Case Series

A total of 7 patients with severe MAC and severe MS were selected to undergo the MATRIX procedure between 2024 and 2025 at 3 large tertiary structural heart disease centers (Morristown Medical Center, Henry Ford Hospital, and William Beaumont Hospital). Institutional review board oversight and waiver from consent were obtained per our institutional policies. Baseline characteristics are summarized in Table 1. The median age of the patients was 78 years, the majority were men (85.7%), with a body mass index of 27.3 kg/m^2^. All patients were in New York Heart Association (NYHA) functional class III or IV with progressively worsening dyspnea. All were not deemed suitable for cardiac surgery. Their imaging characteristics are summarized in Table 1. Patients underwent evaluation with cardiac CTA and transthoracic echocardiogram for procedural planning. CTA demonstrated inadequate MV circumferential calcium for valve implantation with prohibitive risk of LVOT obstruction, precluding TMVR (Supplemental Figure 1). Transthoracic echocardiogram revealed severe MAC and severe MS with elevated peak transmitral gradients of 25 mmHg and mean mitral gradients (mMGs) of 9 mmHg. There was concomitant MR, either mild (71.4%) or moderate (28.6%) in severity. Left ventricular (LV) ejection fraction was preserved at 65%, and there was moderate tricuspid regurgitation in most patients (85.7%).

**Table 1:** Patient demographics and imaging characteristics

Baseline characteristics	
Age, y	78.0 (72.8 – 81.0)
Sex, Female	1 (14.3%)
BMI, kg/m^2^	27.3 (25.8 – 31.5)
NYHA III or IV	7 (100%)
Prior TAVR	3 (42.9%)
Computed tomographic characteristics	
Mitral valve area, cm^2^	4.7 (4.2 – 9.2)
Mitral valve maximum diameter, mm	31.8 (30.6 – 33.0)
Mitral valve minimum diameter, mm	21.0 (19.7 – 29.5)
Echocardiographic characteristics	
Left ventricular ejection fraction, %	65.0 (57.5 – 70.0)
Severe AS, %	1 (14.3%)
Moderate to severe TR, %	6 (85.7%)
RVSP, mmHg	58.5 (49.0 – 70.0)

Continuous variables are presented as median (interquartile range). Categorical variables are reported as n (%).

Abbreviations: AS, aortic stenosis; BMI, body mass index; NYHA, New York Heart Association; RVSP, right ventricular systolic pressure; TAVR, transcatheter aortic valve replacement; TR, tricuspid regurgitation.

## Procedure Details

**Figure 2:**
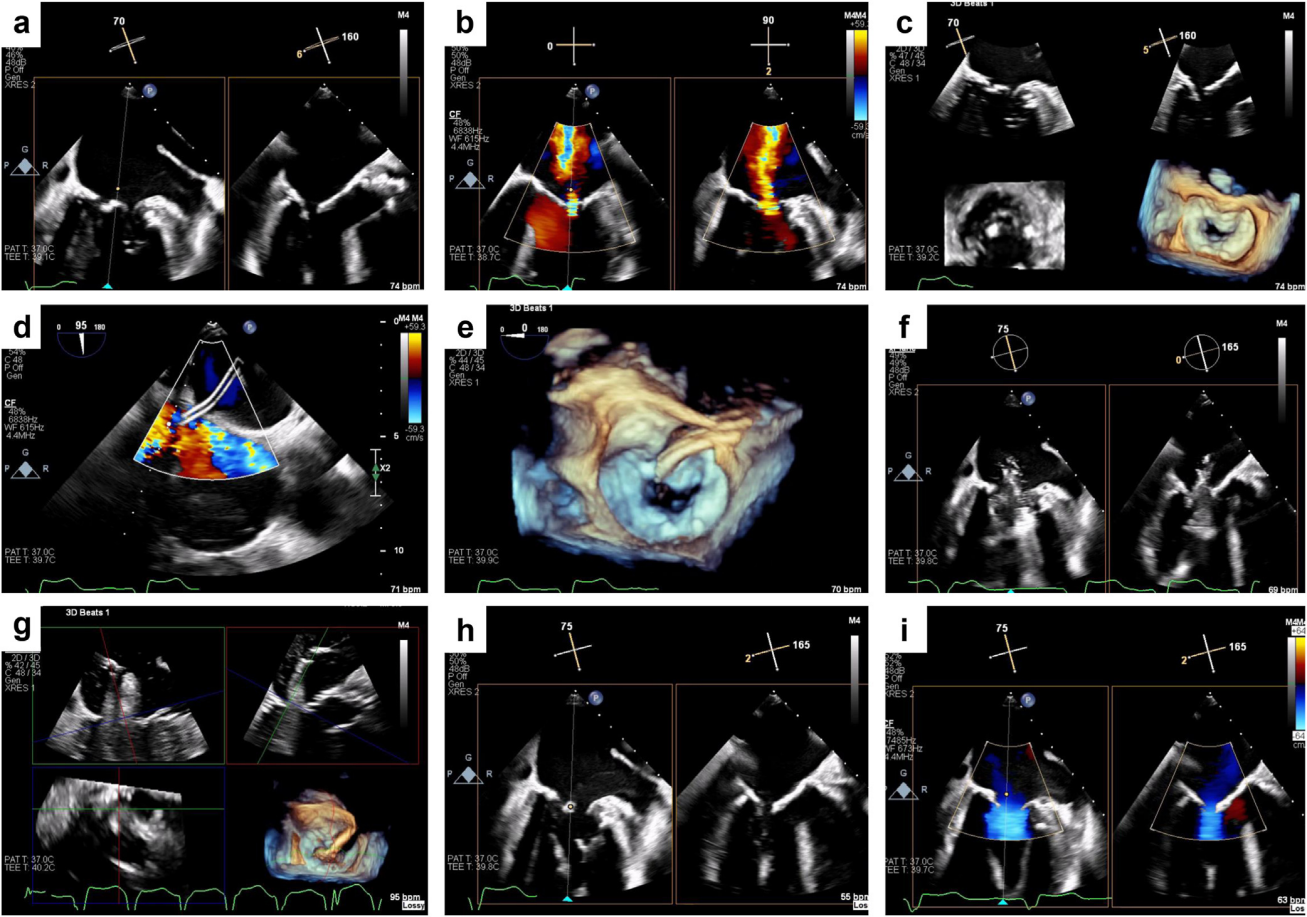
**Representative transesophageal echocardiographic images of the MATRIX procedure. (a)** Severe MS preprocedure on 2D TEE. **(b)** Severe MS preprocedure on 2D color Doppler. **(c)** Severe MS preprocedure on 3D reconstruction. **(d)** Transseptal crossing. **(e)** Alignment of the PASCAL precision system with the orifice of the MV. **(f)** MATRIX on 2D TTE. **(g)** MATRIX on 3D echo. **(h)** Post-MATRIX moderate MS on 2D echo. **(i)** Post-MATRIX moderate MS on 2D color Doppler. Abbreviations: 2D, two-dimensional; 3D, three-dimensional; MATRIX, Mitral Annulo-TRIpsy in eXtreme risk patients; MS, mitral stenosis; MV, mitral valve; TEE, transesophageal echocardiogram; TTE, transthoracic echocardiogram.

**Figure 3:**
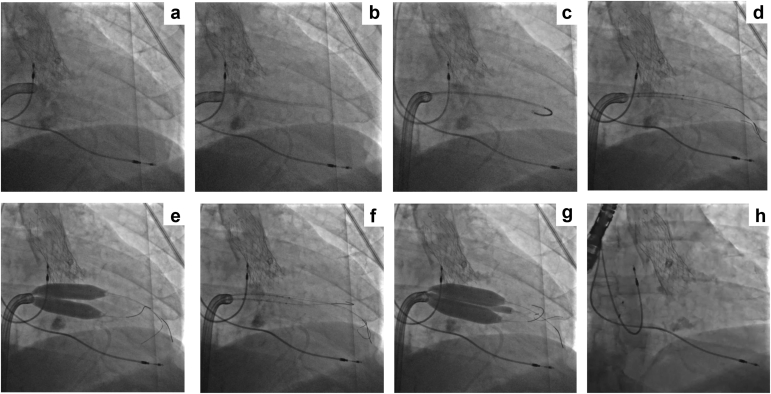
**Fluoroscopic images of the MATRIX procedure. (a)** Steerable PASCAL precision system (Edwards Lifesciences) crossing the intra-atrial septum. **(b)** PASCAL precision system aligned with MV orifice with pigtail catheter that crosses the MV into the LV. **(c)** Pigtail catheter is exchanged for a 6-Fr pigtail catheter (Cordis) in the LV. **(d)** Two guidewires (NITREX, Medtronic; Grand Slam, ASAHI) cross the MV into the LV. **(e)** Simultaneous inflation of two 12 mm shockwave balloons for initial dilation. **(f)** Crossing of the third guidewire across the MV. **(g)** Simultaneous inflation of two 12 mm and one 8 mm shockwave balloons for final valvuloplasty. **(h)** Significant MAC noted on fluoroscopic image. Abbreviations: LV, left ventricle; MATRIX, Mitral Annulo-TRIpsy in eXtreme risk patients; MAC, mitral annular calcification; MV, mitral valve.

A complete equipment list and step-by-step procedural details are summarized in Tables 2 and 3. Procedural characteristics are illustrated in Table 4. The patients were placed under general anesthesia, and a transesophageal echocardiogram (TEE) was performed. Representative TEE images of the MATRIX procedure are summarized in Figure 2. Transfemoral access was obtained, and a guidewire was advanced to the right atrium and positioned toward the intra-atrial septum. The intra-atrial septum was crossed using a standard transseptal technique. A Confida guidewire (Medtronic) was utilized to access the left atrium, which was exchanged for a steerable PASCAL precision system (Edwards Lifesciences, Irvine, California) to align with the MV orifice. A 6 Fr pigtail catheter was advanced across the MV into the LV, followed by 3 guidewires (Nitrex, Medtronic; Grand Slam, ASAHI) to facilitate delivery of the shockwave balloons (Shockwave Medical). Mitral shockwave lithotripsy was performed by inflating 3 balloons simultaneously, followed by delivery of the ultrasonic shockwaves for a full therapeutic cycle of 30 shocks. The size of the IVL balloons is selected based on achieving a degree of oversizing of around 20% above a nominal valvuloplasty balloon (e.g., we would use two 12 mm and one 8 mm IVL balloon if we would have used a 28 mm mitral valvuloplasty [MVL] balloon). Oversizing by 20% above a nominal valvuloplasty balloon will facilitate optimal positioning within the mitral annulus and increase contact with the annulus and subvalvular apparatus when delivering the shockwaves, possibly increasing the efficacy of IVL. Delivery of the ultrasonic shockwaves is repeated multiple times (3 – 5 rounds) until satisfactory results are achieved, allowing blood pressure to recover between inflation of the balloons. Representative fluoroscopic images of the MATRIX procedure are presented in Figure 3. Representative fluoroscopic and echocardiographic videos of the MATRIX procedure may be viewed in Videos 1 – 3.

**Table 2:** MATRIX equipment list

Vascular access	Initial access with a 6 Fr sheath (Pinnacle; Terumo Medical Corporation), exchanged for a 9 Fr sheath (9 Fr 0.038 in x 10 cm; Pinnacle; Terumo Medical Corporation), then exchanged for an 18 Fr sheath (large Check Flo Introducers; Cook Medical). Secondary vascular access with a 6 Fr sheath.
Transseptal crossing	Emerald J-Tip Flex guidewire 0.035 150 cm (Cordis) or Hi-Torque Supra Core guidewire 0.035 190 cm (Abbott) NRG Transseptal Needle 71 cm (Boston Scientific) Confida Brecker guidewire 0.035 260 cm (Medtronic) Edwards PASCAL precision transcatheter valve repair system (guide sheath was used)
MATRIX	Cordis INFINITI 6-Fr Pigtail Catheter 145 110 cm 6SH for wire support (have multiple) Medtronic 0.014 NITREX 300 cm guidewire (have multiple in the room) ASAHI Grand Slam 300 cm Shockwave L6 IVL 12 × 30 mm (inflated to 4 ATM; multiple used) Shockwave M5 IVL 5.0 × 60 mm (inflated to 6 ATM; Shockwave Medical) Shockwave E8 IVL 8.0 × 30 mm (inflated to 4 ATM; Shockwave Medical)
ASD closure (optional)	Abbott’s Amplatzer Cribriform Occluder (or other ASD closure device)
Closure device	Abbott’s Perclose ProGlide and Ethibond suture (or other closure device)

Abbreviations: ASD, atrial septal defect; ATM, atmospheric pressure; IVL, intravascular lithotripsy; MATRIX, Mitral Annulo-TRIpsy in eXtreme risk patients.

**Table 3:** Step-by-step protocol of the MATRIX procedure

1. Obtain vascular access (right and left femoral) and transseptal cross per the usual standard.
2. Steer the PASCAL precision system until aligned with the MV orifice.
3. Advance the 6 Fr pigtail catheter across the MV into the LV.
4. Traverse the MV with 3 guidewires (Nitrex, Medtronic; Grand Slam, ASAHI) in preparation for the initial valvuloplasty.
5. Deliver 2 of the shockwave peripheral balloons of the desired size (12 × 30 mm) over the guidewires, inflate (4-6 ATM was utilized in our procedure for 30 s), then deflate.
6. Deliver the additional shockwave peripheral balloon IVL system of desired size (5.0 × 60 mm), simultaneously inflate (4-6 ATM was utilized in our procedure for 30 s) with the 2 prepositioned shockwave IVL systems (12 mm × 30 mm; 4-6 ATM was utilized in our procedure for 30 s), then deflate.
7. Exchange the third shockwave peripheral balloon IVL system for a large size (8.0 × 30 mm) and simultaneously inflate (4-6 ATM was utilized in our procedure for 30 s) with the 2 prepositioned shockwave IVL systems (12 mm × 30 mm; 4-6 ATM was utilized in our procedure for 30 s), then deflate.
8. Remove all the shockwave IVL balloons and reassess the MV on TEE; maintain guidewire position until acceptable results are achieved.
9. Option to use an ASO device if bidirectional shunting is observed.

Abbreviations: ASO, atrial septal occluder; ATM, atmospheric pressure; IVL, intravascular lithotripsy; LV, left ventricle; MV, mitral valve; TEE, transesophageal echocardiogram; MATRIX, Mitral Annulo-TRIpsy in eXtreme risk patients.

**Table 4:** Procedural characteristics

Number of IVL balloons per patient	
1	0 (0%)
2	1 (14.3%)
3	6 (85.7%)
Maximum diameter of IVL balloons, mm	
8	4 (20%)
10	4 (20%)
12	12 (60%)
Total fluoroscopic time, min	31.0 (21.6 – 40.6)
Cerebral protection device, n (%)	4 (57.1%)

Normally distributed continuous variables are reported as mean ± SD, or as median ± interquartile range if not normally distributed. Categorical variables are reported as n (%).

Abbreviation: IVL, intravascular lithotripsy.

## Results

**Figure 4:**
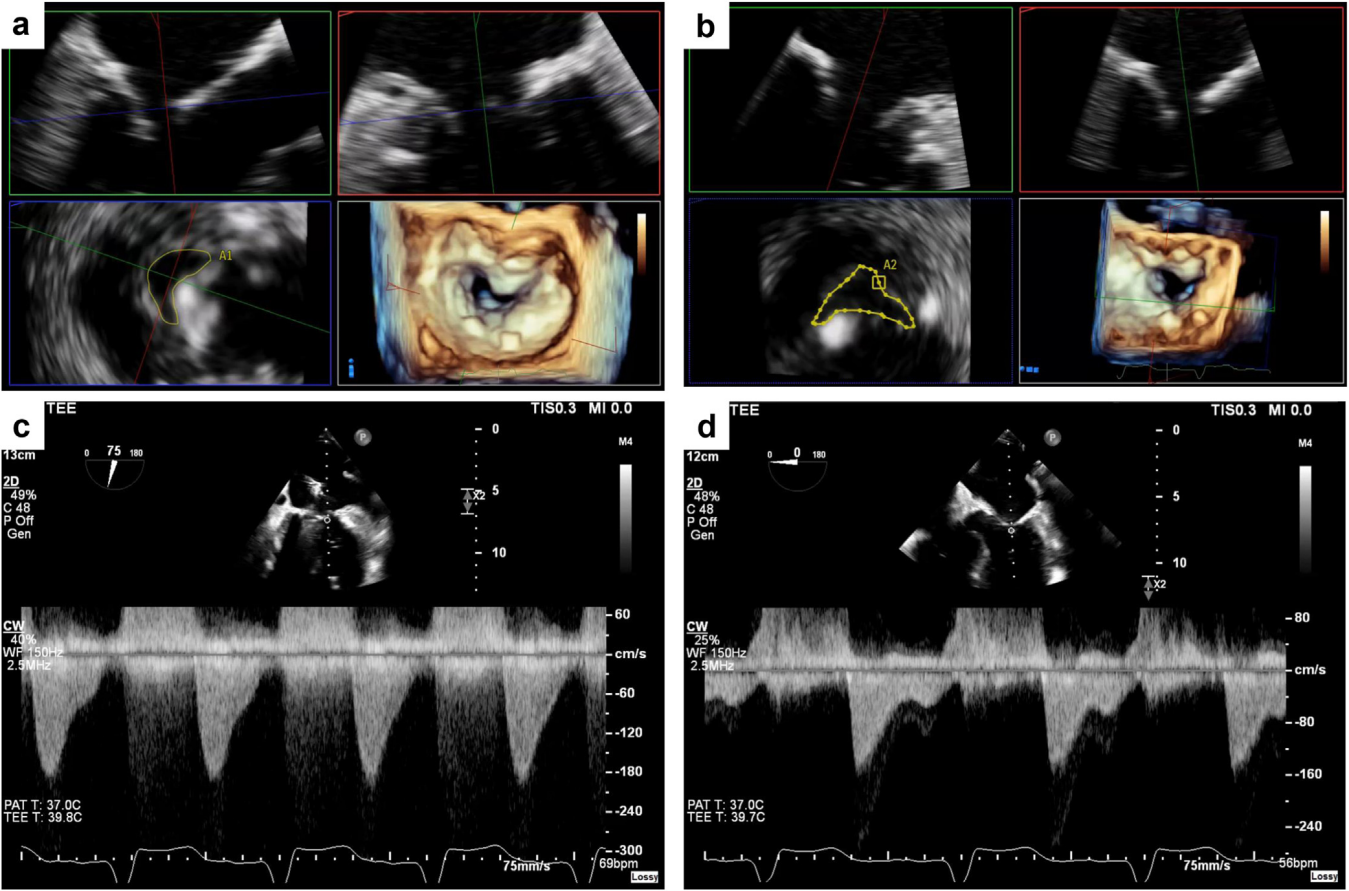
**TEE measurements pre- and post-MATRIX procedure. (a)** Pre-MATRIX procedure with MV area of 1.05 cm^2^ and circumference of 4.9 cm. **(b)** Post-MATRIX procedure with MV area of 1.65 cm^2^ and circumference of 6.8 cm. **(c)** Pre-MATRIX procedure with elevated peak/mean MV gradient of 16/7 mmHg. **(d)** Post-MATRIX procedure with reduced peak/mean MV gradients of 8/2 mmHg. Abbreviations: MATRIX, Mitral Annulo-TRIpsy in eXtreme risk patients; MV, mitral valve; TEE, transesophageal echocardiogram.

**Figure 5:**
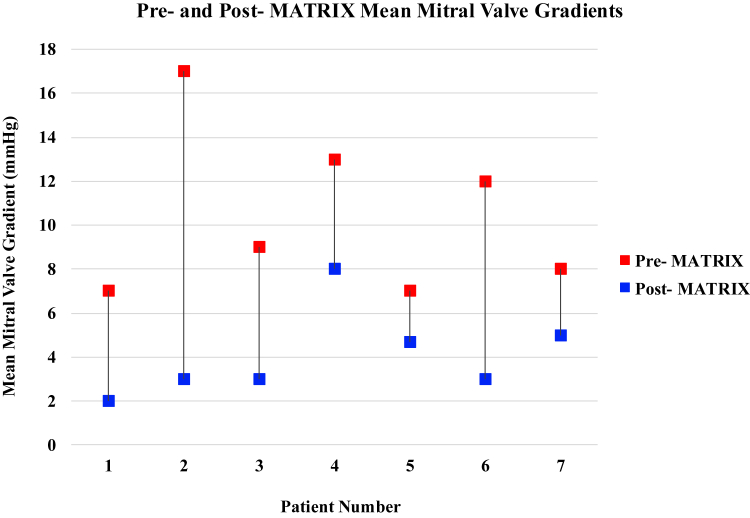
**Pre- and post-MATRIX mean mitral valve gradients.** Pre-MATRIX mMG of individual patients are represented by the red data points. Post-MATRIX mMG of individual patients are represented by the blue data points. Abbreviations: MATRIX, Mitral Annulo-TRIpsy in eXtreme risk patients; mMG, mean mitral gradient.

The transseptal puncture and crossing of the MV were successful in all cases. Procedural characteristics are summarized in Table 4. The median fluoroscopic time was 31.0 minutes. Cerebral protection devices were utilized in 57% of patients. The average number of shockwave balloons used was 2.9 ± 0.4, and the average maximum shockwave balloon diameter was 10.8 ± 1.6 mm. Following the final valvuloplasty, the final mMG assessed with intraprocedural TEE was 3 mmHg, with an absolute difference from baseline of -6.3 mmHg (95% CI 2.6 – 10.1 mmHg; *p* <0.01) (Table 5; Figure 4). Individual changes in mMG are represented in Figure 5. There was no worsening of MR postprocedurally. One patient had a residual mMG of 8 mmHg, and the remaining patients had mMG ≤5 mmHg. A total of 4 out of 7 patients had at least a 50% reduction in MV gradients from baseline. There were no major intraprocedural complications, including death, myocardial infarction, stroke, major bleed, reintervention, or pericardial effusion. In 1 patient, there was a residual atrial septal defect with bidirectional shunting, which was subsequently closed using a 10 mm atrial septal occluder device (Amplatzer Cribriform Occluder; Abbott) without residual interatrial shunting. All patients were discharged alive after the procedure. At day 30 of follow-up, repeat echocardiographic data were available for 6 (85.7%) patients. There were no changes in LV ejection fraction (median 65%; interquartile range: 65-69). A total of 3 (50%) patients remained with mMG <5 mmHg, 2 patients had residual mMG <8 mmHg, and 1 patient had an mMG of 13 mmHg but remained asymptomatic. All patients had either trace or mild MR, which was consistent with the degree of MR prior to the MATRIX procedure.

**Table 5:** Preprocedural and postprocedural echocardiographic characteristics

Echocardiographic parameter	Preprocedure	Postprocedure	*P*-value
Left ventricular ejection fraction, %	65.0 (57.5 – 70.0)	65.0 (65.0 – 65.0)	-
Peak MV gradient, mmHg	25.0 (14.9 – 29.5)	9.0 (7.5 – 22.0)	<0.05
Mean MV gradient, mmHg	9.0 (7.3 – 13.0)	3.0 (3.0 – 5.0)	<0.01
MR severity, %			
None	0 (0%)	0 (0%)	-
Mild	5 (71.4%)	7 (100%)	-
Moderate	2 (28.6%)	0 (0%)	-
Severe	0 (0%)	0 (0%)	-

Normally distributed continuous variables are reported as mean ± SD, or as median ± interquartile range if not normally distributed. Categorical variables are reported as n (%).

Abbreviations: MR, mitral regurgitation; MV, mitral valve.

## Discussion

MS due to MAC is a common valvular pathology with a prevalence of 40% in older adults.^1^^,^^2^ Many patients with severe MAC are at high or prohibitive surgical risk due to multiple comorbidities. Those capable of undergoing SMVR have an associated 6-fold increase in operative mortality related to increased surgical complexity and procedure times.^6^^,^^10^ TMVR may not provide a solution for these patients due to large annular sizes and risk of LVOT obstruction.^9^ Cardiac CTA is an effective modality to measure MAC severity, predict these risks, and determine the feasibility of TMVR.^11^ When TMVR is contraindicated, IVL-PBMV is a suitable alternative for management of severe MAC in these extreme risk patients.

Eng et al. first demonstrated the effective capability of IVL to debulk calcium buildup around the MV annulus prior to PBMV in 2019.^13^ Our group adopted this technique and then performed the largest case-series of IVL-PBMV for severe MAC to demonstrate feasibility.^12^ In a cohort of 24 high-risk, NYHA functional class III or IV, and nonsurgical candidates, the mean MV gradient was successfully reduced from 10.2 ± 3.6 mmHg to 4.8 ± 2.5 mmHg following IVL-PBMV with 100% procedural success and 0% mortality.^12^ However, we were limited to small shockwave balloons with a maximum diameter of 7.8 ± 1.9 mm.^12^ Thus requiring 2-3 balloons to be deployed simultaneously during the procedure despite not fully forming contact with the MV annulus.^12^ Similar findings were recently demonstrated by Alnasser et al.^14^ in a cohort of 15 patients with symptomatic calcific MS. In this study, a single 7 mm IVL balloon was utilized in most patients (93%). A total of 300 shockwave impulses were delivered per MVL. One patient underwent MVL with simultaneous deployment of 2 IVL balloons. Conventional IVL-PBMV risks damaging the native MV annulus via a mechanism of poor contact leading, which could lead to clinically significant increased rates of postprocedural MR. With the development of larger-sized shockwave balloons, we can mitigate these risks and more effectively perform IVL-PBMV utilizing the MATRIX technique.

We successfully performed MATRIX in 7 high-surgical-risk patients with excellent results and without complication. At 30 days post-MATRIX, the transmitral gradients remained low, and the patient reported symptomatic improvement of NYHA class I-II. We demonstrated that use of larger-sized shockwave balloons facilitates contact with the entire MV annulus, which more effectively delivers the acoustic shockwaves to induce calcium fractures and relieve stenosis. A key part of this technique is the use of a large-bore transseptal steerable sheath (i.e., the PASCAL sheath), as it maintains the balloons aligned and centered within the MV annulus and allows the simultaneous delivery of up to three 12 mm balloons. Limitations of MATRIX include operator dependence, commercial availability of the balloons, and center capability. Additionally, we did not characterize in detail the pattern of MAC and whether a differential response to MATRIX exists based on this. Further studies with larger sample sizes are required to validate the safety and feasibility of MATRIX.

## Conclusions

In this preliminary multicenter experience, we demonstrate the feasibility, efficacy, and safety of the MATRIX technique in a cohort of patients with severe MS secondary to MAC. Larger prospective studies in this high-risk patient population are required to validate this new technique. MATRIX offers an alternative treatment option for MAC in those who are at prohibitive surgical risk and where TMVR is contraindicated.

## CRediT Authorship Contributions

All authors made substantial contributions to the conception and design of the study, acquisition of data, analysis and interpretation of data, as well as drafting and editing the manuscript. All authors have read and agreed to the final version of the manuscript.

## Funding

The authors have no funding to report.

## Ethics Statement

Institutional review board oversight and waiver from consent were obtained per our institutional policies.

## Disclosure Statement

G. Giustino is a proctor and consultant for Edwards Lifesciences. K. P. Koulogiannis is a consultant for Edwards Lifesciences and Medtronic. P. Genereux receives institutional research grants from Edwards Lifesciences; consulting fees from 10.13039/100000046Abbott, 10.13039/100006479Cordis, 10.13039/100006520Edwards Lifesciences, Egnite, Haemonetics, 10.13039/100004374Medtronic, Opsens, Puzzle Medical, Pi-Cardia, and 4C Medical; equity in Puzzle Medical and Pi-Cardia; PI of EARLY TAVR trial and PI of PROGRESS trial, both sponsored by 10.13039/100006520Edwards Lifesciences. The other authors had no conflicts to declare.

## Supplementary Material

Video 1 The MATRIX procedure. RAO and cranial view on fluoroscopy of demonstrating 3 × 12 mm shockwave balloons inflated over 3 guidewires.

Video 2 The MATRIX procedure. LAO and caudal view on fluoroscopy of demonstrating 3 × 12 mm shockwave balloons situated within the mitral valve.

Video 3 The MATRIX procedure on transesophageal echocardiography. Transesophageal echocardiogram demonstrating 3 × 12 mm shockwave balloons inflated over 3 guidewires.

Supplemental Figure 1 Distribution of calcification around the mitral annulus. (a-d) Representative computed tomography assessment of the extension, severity, and distribution of calcification of the mitral annulus in a patient with severe calcific MAC. Accumulation of circumferential calcium deposits around the mitral annulus with increased severity in the posterior region with trigone involvement and extension toward the posterior and anterior mitral valve leaflets. Abbreviation: MAC, mitral annular calcification.
