# Supplementary figures and images for: Feasibility, Efficacy, and Safety of the Mitral Annulo-TRIpsy in eXtreme Risk Patients
Source: Struct Heart. 2025 Jun 24;9(8):100683. doi: 10.1016/j.shj.2025.100683 (PMC12399273; doi:10.1016/j.shj.2025.100683)

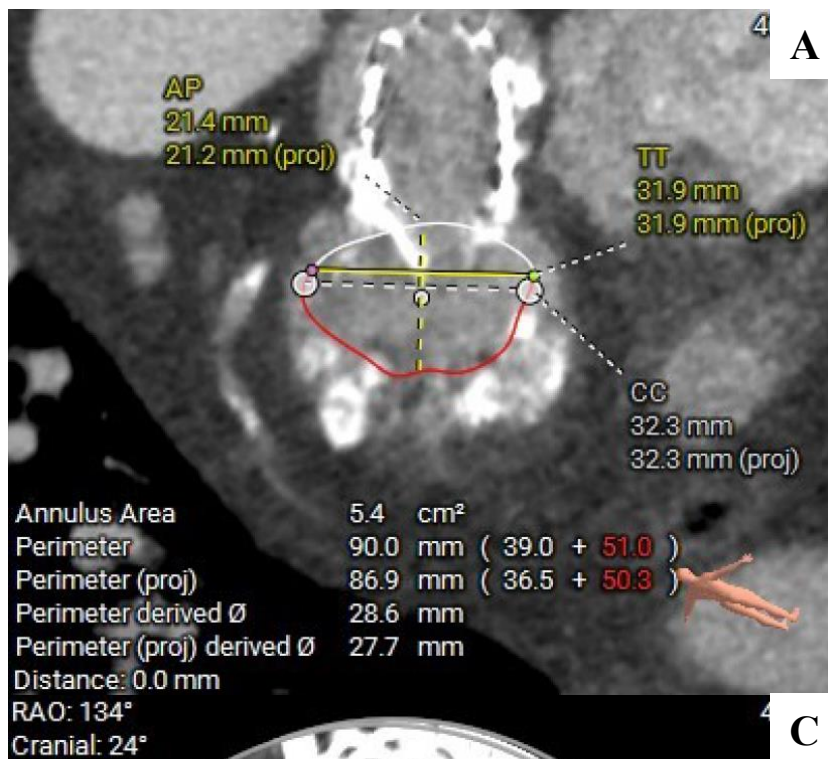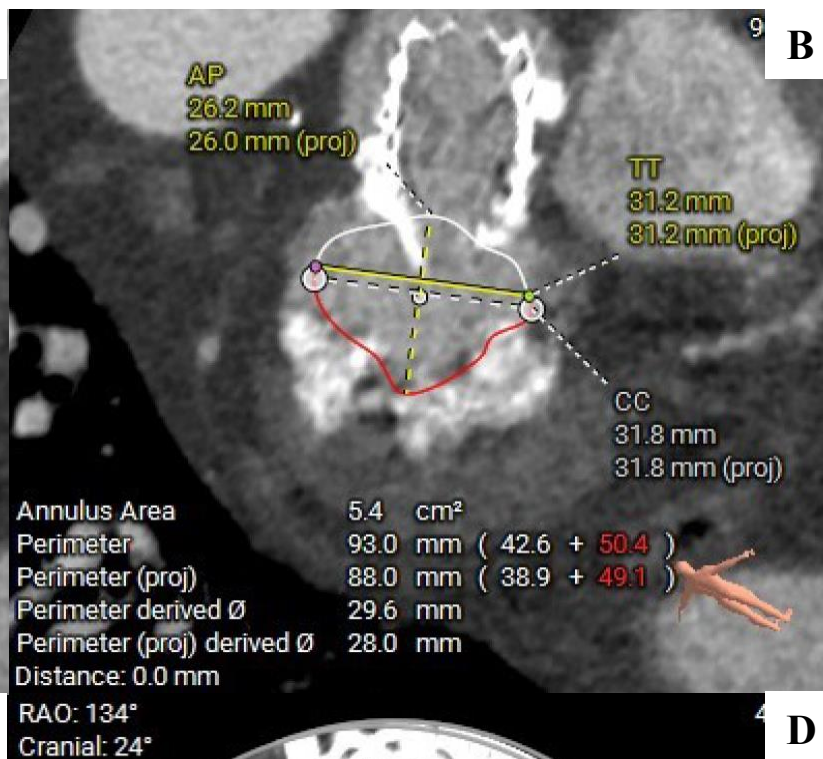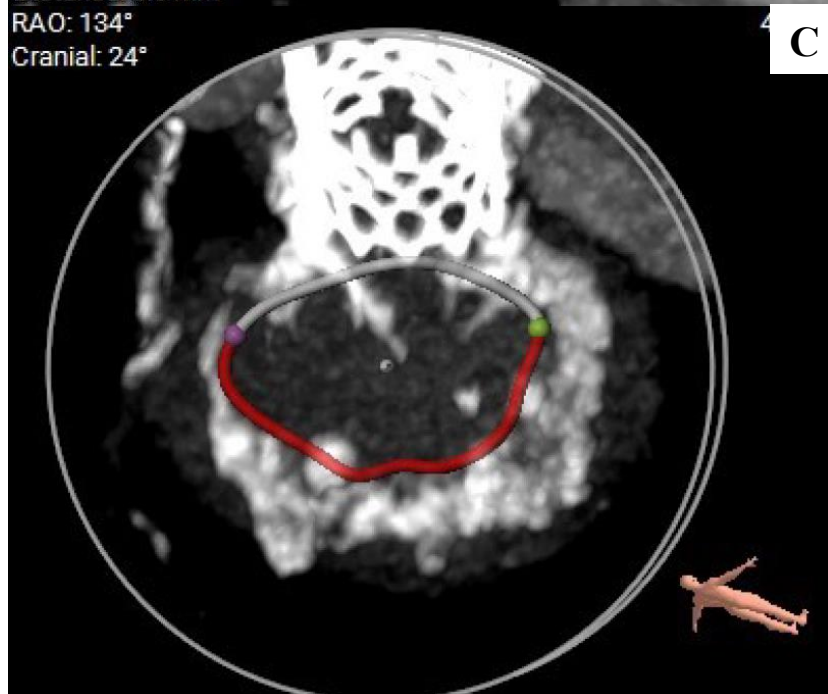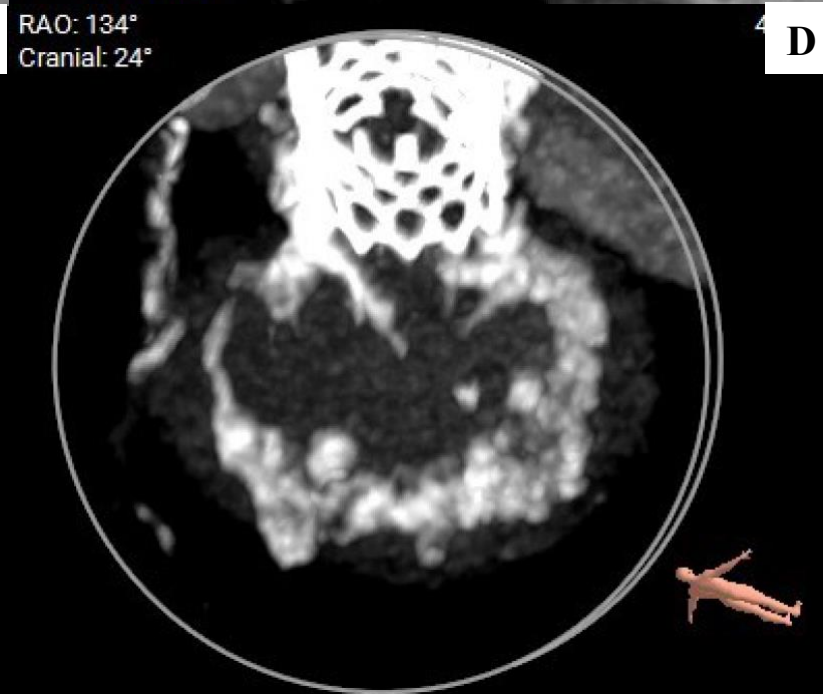

Supplement: Supplemental Figure 1 — Distribution of calcification around the mitral annulus. (a-d) Representative computed tomography assessment of the extension, severity, and distribution of calcification of the mitral annulus in a patient with severe calcific MAC. Accumulation of circumferential calcium deposits around the mitral annulus with increased severity in the posterior region with trigone involvement and extension toward the posterior and anterior mitral valve leaflets. Abbreviation: MAC, mitral annular calcification. [file mmc4.pdf]
